# Spatial Mental Transformation Skills Discriminate Fitness to Drive in Young and Old Adults

**DOI:** 10.3389/fpsyg.2020.604762

**Published:** 2020-12-03

**Authors:** Luigi Tinella, Antonella Lopez, Alessandro Oronzo Caffò, Ignazio Grattagliano, Andrea Bosco

**Affiliations:** Department of Educational Sciences, Psychology, Communication, University of Studies of Bari, Bari, Italy

**Keywords:** interaction regression models, fitness to drive, mental rotation, perspective taking, spatial cognition, driving, ageing

## Abstract

Literature on driving research suggests a relationship between cognition and driving performance in older and younger drivers. There is little research on adults and driving, despite them being the largest age cohort behind the wheel. Among the cognitive domains, visuospatial abilities are expected to be highly predictive of driving skills and driving fitness. The relationship between specific spatial mental transformation skills (i.e., object and self-based ones) and driving performance has not yet been examined. The present study aimed to investigate the relationship between overall cognitive functioning, self and object-based spatial mental transformation skills, and driving performance in a sample of younger and older adult drivers. Participants were comprised of one hundred younger and 83 older adult Italian drivers. Participants completed a computerized driving test assessing traffic stress resilience, visual and motor reaction time, and the ability to obtain an overview of the traffic scenario (DT, vRT, mRT, and ATAV respectively in the Shufried®-Vienna Test System–DRIVESC). The Mental Rotation Test (MRT) and the Object Perspective Taking Test (OPT) were administered in order to assess object-based and self-based spatial mental transformation skills. The Montreal Cognitive Assessment Test (MoCA) was administered control for global cognitive functioning. The effects of education and gender were also controlled in the analysis. The results of the present study suggested that: (1) The effect of age, favoring younger participants, was found in DT, vRT, mRT, and ATAVT tests. (2) The effect of global cognitive functioning was found in DT and ATAV tests. (3) The effect of the spatial mental transformation tests was found in DT, vRT (MRT only), and ATAVT (OPT only) tests. Taken together, these results suggest the specific contribution of spatial mental transformation skills in the execution of complex behaviors connected to the fitness to drive. Prospectively, the results of the present study relating spatial mental transformation skills and driving processes may be a valuable source of knowledge for researchers dealing with the relationship between cognitive resources and navigation aids.

## Introduction

Driving a motor vehicle is one of the most complex, multifaceted, and potentially hazardous tasks that people encounter every day (Michon, 1979; Lee, 2008; Ledger et al., 2019a). It requires the processing of simultaneous environmental cues and the simultaneous execution of several sub-tasks in a safe way (Galski et al., 1992). According to Michon (1979), dangers, such as the risk of being involved in an accident, will occur with some probabilities while driving. A number of factors, acting alone or connected, could cause accidents to occur, such as vehicle (i.e., tires’ conditions), road (i.e., road grip), environmental (i.e., weather conditions), and human (i.e., cognitive failures, attentional blinks) factors (Treat et al., 1979; Evans, 1991; Eboli and Forciniti, 2020). Human factors, which are considered the cause of more than 70% of road accidents (Evans, 1996; Petridou and Moustaki, 2000; Istat, 2019), refer to an individuals’ driving skills, driving style, cognitive abilities, and personality measures (Evans, 1991; Elander et al., 1993).

According to Elander et al. (1993), driving skills refer to strengths and weaknesses of the individual’s driving performance, that is the execution of the ensemble of driving’s sub-tasks (e.g., steering wheel, time taken to react to dangers) that improves with practice. The driving style includes the way people choose to drive based on habits consolidated over years (e.g., speed or proneness to violation), influenced by attitudes toward driving and the individual’s values. Driving skills and driving style correspond to what Evans (1991) initially theorized as “driving performance” and “driving behavior,” respectively. Both measures are considered *intrinsic* to driving (Elander et al., 1993). In addition to this, Elander et al. (1993) also proposed a framework of psychological factors related to crash rates that included measures that are *extrinsic* (but related) to driving. For example, cognitive abilities (i.e., reaction times, visual attention) and personality traits (i.e., type A personality, sensation seeking) would count among a driver’s extrinsic measures.

More specifically, general cognitive functions, considered accurate predictors of driving performance, include attention, executive functioning, memory, logic reasoning, psychomotor abilities, and visuospatial skills (Reger et al., 2004; Uc et al., 2006b; Sommer et al., 2008; Mathias and Lucas, 2009; Aksan et al., 2015; Overton et al., 2015; Zicat et al., 2018; Palmiero et al., 2019).

Each driving sub-task requires a combination/integration of cognitive domains, which have to process a wide range of sensory information in order to safely manage the vehicle and to deal with hazards. For example, using the steering wheel to track the road requires visual tracking, psychomotor abilities (i.e., oculomotor coordination), praxic abilities (i.e., grasping), and processing speed.

Visuospatial skills can be considered as *classics* among the cognitive domains contributing to driving (Sommer et al., 2008). Indeed, driving requires the ability to clearly see the shape and color of objects and reliably estimate their distance in order to rapidly and effectively decide to perform driving maneuvers (Galski et al., 1992; Groeger, 2000). Measures of visual acuity, visual attention, motion detection, visuo-constructive abilities, and immediate visuo-spatial memory are considered good predictors of real-world driving (for a review see Anstey et al., 2005; see also Mathias and Lucas, 2009). Moreover, driving requires selecting routes, maintaining the path while monitoring one’s own position with respect to the goal location, and selecting and recognizing routes and places (e.g., Golledge, 1999). Visuospatial skills, other than to supplement driving performance, also support human navigation. In particular, spatial mental representation skills are considered predictive of the ability to navigate (Kozhevnikov et al., 2006; Muffato et al., 2017; Ruginski et al., 2019). Despite this, the specific contribution of representation and transformation of spatial information has been neglected in driving research. In a recent study, Nori et al. (2020) demonstrated that a specific strategy of spatial navigation predicted self-reported driving behaviors. The authors found that good navigators who preferred survey strategy for spatial navigation reported fewer driving violations and less aggressive behavior than route and landmark strategies’ users.

Several studies outlined that road crashes are partially determined by individual factors unrelated to safe driving (Evans, 1991; Fox et al., 1998; Sommer et al., 2008). For example, several personality traits have been demonstrated to contribute to effective driving behaviors (Sommer et al., 2008) and showed predictive validity on measures of driving fitness (Oltedal and Rundmo, 2006). In particular, personality traits seem to influence decisions about the goals to achieve while driving (Sommer et al., 2008). For example, speeding is a driving behavior linked with Sensation Seeking (Whissell and Bigelow, 2003). Moreover, the chosen driving speed is one aspect of driving style that has been demonstrated as being determinant for a differential crash-involvement (Elander et al., 1993). In other words, driving styles seem to be an expression of both cognition and personality that, in turn, influence behavior and the fitness to drive.

### The Assessment of Fitness to Drive

Fitness to drive (FTD) is defined as “the ability to drive safely without problems caused by physical abilities, injuries, medical or mental health” (Road Safety Canada Consulting, 2011).

The assessment of FTD requires steady and accurate monitoring of the drivers’ level of competencies in order to meet the medico-legal demands for license-renewal and relicensing. The background research on the evaluation of FTD suggests that the performance-based assessment offers great clinical validity in predicting driving performance (Dickerson et al., 2011). The assessment of FTD includes on-road tests, simulated driving tests, and cognitive evaluation (Galski et al., 2000; Caffò et al., 2020). The on-road test is considered the *gold-standard* in measuring driving performance (Mathias and Lucas, 2009). Despite this, it is widely shared that the high costs and potential risks associated with on-road tests has limited their diffusion as a research task (Lee et al., 2003; Mathias and Lucas, 2009).

Conversely, literature shows moderate agreement in supporting the validity of driving simulators for the assessment of FTD with respect to neuropsychological and on-road tests (Caffò et al., 2020). Several studies showed that driving simulators are valid substitutes for on-road driving tests (Lee et al., 2007; de Winter et al., 2009) and for laboratory testing (i.e., cognitive assessment; Crizzle et al., 2012; Murray, 2017). Despite this, the lack of research on the standards and psychometric properties of driving tests, together with the phenomenon of simulation sickness, limited their use (Caffò et al., 2020). The cognitive assessment includes both the use of paper-and-pencil and computerized tests. Paper and pencil cognitive evaluation should not be used alone to make relicensing decisions (e.g., Brooks and Hawley, 2005). Moreover, there is no broad agreement on the cognitive domains to be assessed and tests to be employed. Despite these two limitations, the cognitive assessment through psychometric standardized tools is a cost-effective method to identify unsafe drivers who need further driving assessment (e.g., McKenna et al., 2004) and to detect some potential impairments undetectable through neither the on-road test nor through the simulated drive (e.g., Anstey et al., 2005). Tests most frequently used for assessing driving skills in both on-road and simulated driving are: Trail Making Test, Wechsler Adult Intelligent Scale subtests, Rey Complex Figure Test, Mini Mental State Examination, Useful Field Of View, Benton Line Orientation, and Clock Drawing Test (for a review see, Anstey et al., 2005; see also, Mathias and Lucas, 2009). Computerized cognitive assessment provides an evaluation of cognitive, perceptive, and motor functions related to driving. The DRIVESC® package of the Vienna Test System (Schuhfried GmbH, 2016) is an example of such an assessment. This tool is generally used in clinical context for the assessment of FTD in healthy drivers and the improvement/training of the impaired sensorimotor and cognitive prerequisites due to injuries and disease. The DRIVESC includes three sub-tasks: (a) the Determination Test (DT), a complex reaction task which evaluates an individual’s traffic stress resilience; (b) the Reaction Test, a sort of *go no-go* (simple reaction) task which provides measures of speed reaction, motor speed, and inhibitory control; and (c) the Adaptive Tachistoscopic Traffic Perception Test (ATAVT), a visual attention task which evaluates an individual’s ability to obtain an overview of static traffic visual scenes. These single subtests showed significant correlations with on-road driving measures in a sample of active drivers aged from 19 to 91 years (Schuhfried GmbH, 2016), as well as predictive validity in discriminating the driving performance of healthy older drivers from those with traumatic brain injury and stroke (Vetter and Debelak, 2012). It has been previously demonstrated that computer-based tools ensure test administrator’s independence and are preferable with respect to paper and pencil tests (Kubinger, 1995). Finally, this tool provides driving-related measures that avoid simulation sickness-related issues.

The literature on FTD showed that the largest part of the research on FTD reported comparisons between young and elderly drivers, providing a limited view on driving performance in other age ranges (Svetina, 2016). The comparison between young and elderly drivers is methodologically beneficial to investigate specific/differential features of older and younger drivers. Yet changes in cognitive functioning could also occur from 30-years-old (Salthouse, 2009). Since cognitive functioning constantly changes after the age of 30, adult drivers should be included in research, thus shifting the focus to the comparison between young and adult drivers. This becomes more important when considering that the onset of the neurodegenerative process that leads to dementia begins about 10–12 years before the diagnosis (Sperling et al., 2011), largely overlapping with active driving ages. Moreover, measures of overall cognitive functioning collected by tests on mental status have been widely employed in research focused on > 65-year-old drivers, but scarcely in studies regarding younger drivers. In a recent study, Ledger et al. (2019b) showed that measures of mental status significantly predicted the overall driving performance in a sample of middle-aged drivers only.

Despite the abovementioned attention devoted to driving-related visuo-spatial skills approaching the predictive validity of several visuo-spatial computerized and paper-and-pencil tests (for a review, Mathias and Lucas, 2009), little is known about the relationship between basic *spatial mental transformation processes*, namely—*mental rotation* and *spatial perspective taking*—and measures of FTD. Basic spatial mental transformations skills involve the ability to imagine/monitor the movement of 3-dimensional objects in the space (i.e., object-based transformations) or to mentally change the personal perspective (i.e., egocentric/self-based transformations). Object-based and self-based spatial mental transformations, often measured by using the Mental Rotation Task (Vandenberg and Kuse, 1978) and Perspective Taking Task (Kozhevnikov and Hegarty, 2001), play a key role in mentally transforming spatial/environmental information and are related to spatial updating (Wolbers and Hegarty, 2010; Wolbers and Wiener, 2014). These two spatial abilities are important for human navigation (Ruginski et al., 2019) when exploration is based on walking. Recently, Ruginski et al. (2019) demonstrated that long-term GPS use negatively affected mental rotation and perspective taking skills that, in turn, were found to be associated with environmental learning outcomes (Muffato et al., 2017). Mental rotation and perspective taking skills should gain importance also in navigation by car, supporting complex behaviors connected to driving when speed is significantly higher and therefore decision times must decrease. To the best of our knowledge, there are no studies that have investigated the direct relationship between these two spatial mental transformation skills and the FTD.

Finally, it is unclear whether and how the interaction within the demographic variables (i.e., age, gender, and education) and between demographic and cognitive variables affects the FTD.

### Demographic Factors Affecting Fitness to Drive

When reference is made to human factors, it is necessary to consider others key human characteristics, useful for the identification of high-risk drivers, namely: age, gender, and level of education (Lourens et al., 1999). Regarding age, both young and adult/elderly drivers are involved in traffic accidents (Prendergast, 2012; Oster and Strong, 2013; Fraade-Blanar et al., 2018; Castellucci et al., 2020). The increase of older drivers has been demonstrated to have had specific consequences on the traffic stream, especially at intersections (e.g., an increase in start-up lost time; Lu and Pernía, 2000). According to Salthouse (2009), cognitive functions accomplish their full maturation in early adulthood, gaining a peak between the range of age 22–27, then start a slow decline after age 30 that accelerates around 60 years and over. It is known that with the increase of age, some medical conditions, such as neurological damage/disease, visual problems, or cognitive impairment, could occur (Ball and Owsley, 1993; Vernon, 1995; Reger et al., 2004; Whelihan et al., 2005; Mathias and Lucas, 2009). However, in a recent study, Ledger et al. (2019a) found that measures of cognitive functioning similarly predicted simulated driving performance in young and mature/elderly drivers, concluding that cognitive functioning affected driving performance throughout life. The incomplete maturation of cognitive functions in young-adulthood and the cognitive decline early observed in mature adults and accelerated in the elderly can cause similar quotes of crash rates (Ledger et al., 2019a). Moreover, young drivers seemed to be more prone to committing driving violations due to cognitive errors (i.e., inhibitory control and decision making—Pharo et al., 2011), behavioral habits (i.e., speeding—Parker et al., 1995; Williams and Shabanova, 2003), and personality disposition (i.e., trait anxiety and sensation seeking—Cestac et al., 2011; Zicat et al., 2018), breaking the rules more frequently (Simon and Corbett, 1996; Cordellieri et al., 2016) compared to the elderly.

Gender differences in driving literature were established at both behavioral and cognitive levels (Getzmann et al., 2018). Males showed less concern for driving safety issues (Butters et al., 2012) and higher speed driving (Hagen, 1975; Taylor et al., 1991; Lourens et al., 1999), had a greater tendency to take risks (Evans, 1991), a greater likelihood to commit driving violations (Rhodes and Pivik, 2011), and more aggressive behavior and proneness to being involved in road accidents than females (Simon and Corbett, 1996; Jiménez-Mejías et al., 2014). Cordellieri et al. (2016) demonstrated that young male drivers were more prone to accept driving violations, speeding, and alcohol and drugs use. Females show greater stress vulnerability while driving than males (Matthews et al., 1999). Males also showed better performance than females in simple, but not in complex, reaction tasks (Dykiert et al., 2012).

Finally, the role of education in predicting driving outcomes is less clear. Hemenway and Solnick (1993) showed that richer and more educated drivers were more confident when reporting their driving habits, such as speed used, than drivers with lower levels of socio-economic status and education. According to Shinar et al. (2001), education constitutes a strong cognitive component of safe driving attitudes. These authors tested the hypothesis that income was related to compliance with social norms among the most educated people. Results showed that the tendency to speed decreased with age and increased with education and income, whereas the use of safety-belts increased with age and education but not with income.

### The Present Study

The present study aimed to investigate whether demographic and cognitive variables predicted FTD in two age groups, evaluating the specific contribution of both *object-and self-based spatial mental transformations*. It was assumed that the performance in driving tasks would be predicted by (1) age, gender, and the level of education; (2) the overall cognitive functioning; and (3) both the measures of spatial mental transformation. Considering the above-mentioned literature, a negative effect for age group is expected on driving prerequisites (e.g., Salthouse, 2009; Svetina, 2016), while a positive effect is expected for cognitive functioning (e.g., Mathias and Lucas, 2009; Ledger et al., 2019a). The last hypothesis is supposed to be relevant since all the driving tasks used here involve (at least) spatial visualization skills. Mental transformations of spatial information, which are demonstrated to be relevant for human navigation (e.g., Wolbers and Hegarty, 2010) and environmental learning (Ruginski et al., 2019), may also be of relevance in supporting driving performance. It was decided to use the driving-related measures as separate outcomes rather than collapsing them into a single measure of fitness to drive in order to investigate the specific contribution of each predictor on each driving prerequisite. Moreover, the role of first order interactions between demographics (i.e., age, gender, and level of education) and cognitive predictors (i.e., overall functioning and spatial transformation skills) was assessed in the attempt to better predict driving measures. Consequently, the best fitting model was retained after the comparison between the main effect and the first order interaction model.

## Materials and Methods

### Participants

A power analysis to estimate the sample size was carried out using G^∗^Power 3.1 (Faul et al., 2009), with the following parameters: a p level of 0.05, a cautious low effect size (0.12), and a power of 0.80. Results indicated that a sample size of 120 participants was sufficient to warrant an 80% chance of correctly rejecting the null hypothesis.

One hundred eighty-three healthy participants, 63 females, between 18 and 64 years of age, took part in the study. One hundred younger adults (i.e., 33 females, age M ± SD = 23.1 ± 3.55; level of education M ± SD = 13.2 ± 1.06, years) and 83 older adults (i.e., 30 females, age M ± SD = 54.1 ± 7.29; level of education M ± SD = 11.3 ± 2.60, years) were enrolled in the study. All participants were required to: have Italian as their mother tongue; hold a valid current driver’s license, provisional or above; have normal or corrected to normal vision; have driven more than one time within the last month; and not be or had been a professional driver (e.g., taxi driver, truck driver, transporter on delivery, etc.). Descriptive statistics for the two groups are reported in Table 1. The participants, blind to the hypothesis of the study, were volunteers recruited with the support of a proxy informant, generally undergraduate and graduate students, trainees, and employers of the Department. All participants signed their informed consent prior to the enrolment in the present study.

**TABLE 1:** Correlation matrix between the variables.

	AGE	GEN	EDU	LICYR	DRIF	MOCA	MRT	OPT	DT	RTM	RTV	ATAVT	*M*	*SD*	Chronbach’s α
AGE	−												37.7	17.1	−
GEN	0.05	−											−	−	−
EDU	−0.38***	–0.01	−										12.3	2.1	−
LICYR	0.95***	0.09	−0.27***	−									17.3	15.8	−
DRIF	0.21**	0.10	–0.07	0.19	−								3	2	−
MOCA	−0.24***	0.00	0.09	−0.23**	–0.11	−							25.0	2.8	0.56
MRT	−0.40***	0.27***	0.15	−0.39***	–0.07	0.34***	−						18.4	9.4	0.89
OPT	0.41***	–0.18	−0.20**	0.39***	0.02	−0.41***	−0.54***	−					74.1	55.0	0.83
DT	−0.64***	–0.04	0.20**	−0.61***	–0.09	0.38***	0.44***	−0.50***	−				66.0	24.1	0.96
RTM	−0.39***	0.45***	0.12	−0.35***	0.00	0.18	0.35***	–0.32	0.30***	−			48.9	25.6	0.97
RTV	−0.32***	0.19**	0.11	−0.32***	0.00	0.18	0.30***	–0.18	0.37***	0.52***	−		53.0	27.2	0.91
ATAVT	−0.45***	0.03	0.15	−0.48***	0.00	0.37***	0.33***	−0.39***	0.48***	0.27***	0.32***	−	51.5	28.0	0.62

Mean (M) and Standard Deviation (SD) for each variable. Median (Mdn) and Inter-quartile Range (IQR) are reported for the ordinal variable (DRIF); reliability coefficients are reported. **p < 0.01, ***p < 0.001.AGE, age in years; GENDER, gender of participants; EDU, years of education; LICYR, years of driving license; DRIF, driving frequency; MOCA, MoCA corrected score; MRT, Mental Rotation Test score; PT, Perspective Taking Test score; DT, Schuhfried Vienna “Determination Test” score; RTM, Schuhfried Vienna “Motor Reaction Times” (msec.); RTV, Schuhfried Vienna “Visual Reaction Times” (msec.); ATAVT, Schuhfried Vienna “Obtaining an Overview” score.

The Ethical Committee of the Department of Education, Psychology, and Communication approved the study protocol, and the whole study was performed following the Helsinki Declaration and its later amendments.

### Materials and Procedure

All participants were from the metropolitan area of Bari, Italy. Participants had to be in a good general state of physical and psychological health. All participants were enrolled between March and June 2019. A brief interview was administered by supervised trainees in neuropsychological assessment to collect demographic information, to exclude neurodegenerative and vision/hearing disorders, and to gather information about participant’s driving habits and years of license. The frequency of driving was recorded as an ordinal variable (1 = “2/3 times per month”; 2 = “at least once a week”; 3 = “more than one time per week”). After completing the interview, all participants completed the following tests according to the reported order:

#### Measure of Overall Cognitive Functioning

Overall cognitive function was assessed through the Montreal Cognitive Assessment (MoCA; Nasreddine et al., 2005), which evaluates several cognitive domains, namely visuospatial/executive, naming, attention, language, abstraction, memory, and orientation on a 30-points scale The best cut-off used in an Italian sample was a MoCA score = 17 (Bosco et al., 2017, 2020) for discriminating participants with probable cognitive impairment. No one was excluded from the sample.

#### Measures of Spatial Mental Transformation

The *Mental Rotation Test* (MRT) is a paper-and-pencil test of spatial visualization and object-based spatial mental transformation. MRT was developed by Vandenberg and Kuse (1978) based on the stimuli used initially by Shepard and Metzler (1971). The test contains 20 items (two-dimensional drawings of three-dimensional objects) and each item consists of a criterion figure, two correct alternatives, and two distractors. Correct alternatives have an identical structure to the criterion but are shown in a rotated position. Participants must select the correct alternatives. Each line is considered correct if both choices are correct. The test is divided into two parts, with 3 min to accomplish each part. The test administration includes the execution of three trials after the task’s explanation and before starting the test. The entire procedure takes about 10 min.

To assess participant’s perspective taking abilities, a revised form (Hegarty and Waller, 2004) of the *Object Perspective Taking Test* (OPT; Kozhevnikov and Hegarty, 2001) was administered. OPT is a paper-and-pencil test that examines self-based egocentric spatial mental transformation skills. For each of the 12 items, a configuration of seven objects was drawn on the top half of a sheet paper. Participants were asked to imagine being at the position of one object in the configuration, facing another object, and to indicate the direction to a third object (the target). A circle was drawn in the bottom half of the sheet paper, the imagined standing point was drawn in the center of the circle, and the imagined heading (direction to the second object) was represented by an arrow pointing vertically up. Participants were asked to draw an arrow from the center of the circle pointing in the direction of the target object. The item’s score was the absolute directional error, namely the deviation in degrees between the participant’s answer and the correct direction to the target. The total score is the average deviation across items. A high total score corresponds to a lower level of ability in OPT. The time limit to accomplish the test was 5 min. The test administration includes the execution of one trial after the task’s explanation and before starting the test. The entire procedure takes about 10 min.

#### Fitness to Drive Screening

The Fitness to Drive Screening (DRIVESC-Version 03; Schuhfried GmbH, 2016) test set is part of the Shuhfried’s Vienna Test System. It was used to test the participants’ fitness to drive. DRIVESC test set has already been shown in several studies to have predictive validity on real-world and simulated driving performance (Schuhfried and Prieler, 1997; Schuhfried, 1998; Karner and Neuwirth, 2000; Kristöffl and Nechtelberger, 2001; Vetter and Debelak, 2012). The criterion validity of the DRIVESC test set was measured as the point-biserial correlation between the subtests and a standardized driving test and estimated by using additive regression analysis (see Vetter and Debelak, 2012). The study of Vetter and Debelak (2012) showed an additive validity equal to rpb = 0.41. The administration was consistent with the use of a standard computer test that guarantees the test-administrator independence, reliability of interpretation, and security against miscalculation of the measures. The apparatus included an LCD computer monitor (19 in wide), a standard audio output device (headset), an ergonomic response panel, and two-foot pedals. The experimental screening took approximately 25 min to complete the three different subtests: Reaction Test (RT), Determination Test (DT), and Adaptive Tachistoscopic Traffic Perception Test (ATAVT).

∙ The RT involves the ability to respond to specific auditory and visual stimuli as quickly and accurately as possible. It provides two distinct measures: (i) Reaction speed (visual reaction time; RTV), that is, the amount of time between the onset of the stimulus and the start of the response movement and (ii) Motor speed (motor reaction time; RTM), that is the time between the moment in which the participant’s finger leaves the rest button and the moment in which the reaction button is pressed. Both these measures are taped in milliseconds where a short-time reaction (i.e., high visual and motor reaction speed) corresponds to a higher ability to quickly respond and carry out the planned action sequences in relevant stimulus-reaction situations.

∙ The DT provides a measure of reactive stress tolerance, that is, the individual’s ability to react quickly and accurately under stress. It includes five optical stimuli of different colors, two different acoustic stimuli, and two visual signals for the foot-pedal keys (respectively, left and right). Participants had to press the corresponding button or foot pedal as quickly and accurately as possible. The software varies the speed of stimuli presentation based on the respondent’s ongoing performance through a computer adaptive system and records participants’ performance in terms of accuracy (i.e., hits, omissions, and false alarms) and response delay (i.e., milliseconds).

∙ The ATAVT measures the individual’s ability to rapidly identify objects and visual patterns (Carroll, 1993) potentially relevant for an overview of the traffic visual scene. ATAVT was administered in the *right-hand traffic form* according to the Italian Traffic Laws. In this subtest, pictures of traffic scenarios are presented for 1 s after an acoustic cue. After each picture, the respondent is requested to tell if one or more elements were present in the picture, choosing among listed options (i.e., motorcycles/bicycles, automobiles, traffic signs, traffic lights, and pedestrians). The participants’ performance is judged based on the number of correct responses (omissions and false alarms are also recorded). The scores of the three subtests are provided as a raw score (called *parameters*) and percentile ranks. For the statistical analysis only percentile ranks have been considered.

The entire procedure was made clear to the participants beforehand. Participants were assessed individually in a silent and well-lit room, without disturbances, in the Department of Psychology of the university. Each assessment session was accomplished by two instructed research assistants and lasted 60–90 min, with breaks provided as requested by participants.

### Data Cleaning and Transformation

Three group of variables were considered for the statistical analysis: the demographic measures (i.e., age, gender and education), the cognitive measures (MoCA, MRT, and OPT), and the fitness-to-drive measures (i.e., DT, RTV, RTM, and ATAVT). Before running the analysis, data were controlled for missing data and outliers examining box plots. No missing data were found. Normality, Linearity, and Homoscedasticity were also assessed. Linearity was assessed calculating the Variance inflation Factors (VIF). Homoscedasticity/Heteroscedasticity (the comparison between variances of variables) was examined from bivariate scatter plots. Since some variables were differently skewed, all continuous measures were transformed into their square roots. This transformation was also used to reduce the impact of outliers (Tabachnick and Fidell, 2019). After the transformations, no outliers were found, and the distribution of all variables deviated from normality (Shapiro-Wilk, *p* < 0.001).

### Statistical Analysis

A Chi-squared test between gender (females and males) and age (younger and adults) variables was performed in order to test for their independence. Independent samples *t*-tests were performed to test for differences in performance measures between both age and gender groups. An independent sample *t*-test was performed in order to test differences in education degree between younger and older and adults. A series of ANOVAs were performed to test for differences in the driving prerequisites’ performance among the reported driving frequency. Correlation coefficients and related *p*-values were calculated between all the variables.

Moreover, reliability analysis was performed for those measures: MoCA, MRT, OPT, DT, RT, and ATAVT. Finally, in order to investigate the influence of demographic and cognitive variables on driving performance, a series of multiple regression analyses was performed using the software R, lavaan package (Rosseel, 2011); the value of p was set to 0.05 for calculation of statistical significance. Two models for each measure of FTD performance as criterion were tested, considering as predictors: a) the demographic and the cognitive variables’ main effects and b) the main effects and first order’s interactions of such predictors. The effect size Cohen’s f^2^ was estimated for each model (Selya et al., 2012).

## Results

### Descriptive Statistics and Preliminary Analyses

Table 1 shows means, standard deviations, and correlation coefficients for the variables employed in this study. Cronbach’s α is reported for the psychometric standardized tools. The means and standard deviations of the tasks for both age and gender groups are reported in Supplementary Table S1. The results of Chi-squared test on contingency tables indicated that, in the composition of the sample, gender was not associated with age (χ^2^ = 0.199; *p* = 0.66). The results of *t*-test for independent samples revealed a significant difference in terms of education degree between the younger and older adults [EDU: *t*(181) = 5.95; *p* < 0.001] with a large effect size (Cohen’s *d* = 0.88), favoring the young. Considering the driving frequency, seven participants reported to drive two/three times per month (3.8%), 12 participants responded to drive at least once a week (6.5%), and the largest part of the sample reported to drive more than one time per week (124 participants; 67.4%). No significant differences were found in the DRIVESC subtests performance among the different classes of time of driving [DT: *F*(2, 180) = 1.21, *p* = 0.30; RTM: *F*(2, 180) = 0.61, *p* = 0.54; RTV: *F*(2, 180) = 1.02, *p* = 0.36; ATAVT: *F*(2, 180) = 0.17, *p* = 0.84]. Moreover, *t*-tests for independent samples revealed significant differences between the two age groups in cognitive and driving tests, in favor of the young [MoCA: *t*(181) = 2.90, *p* < 0.005; MRT: *t*(181) = 5.49, *p* < 0.001; OPT: *t*(181) = −5.85, *p* < 0.001; DT: *t*(181) = 9.87, *p* < 0.001; RTM: *t*(181) = 5.24, *p* < 0.001; RTV: *t*(181) = 4.29, *p* < 0.001; ATAVT: *t*(181) = 5.94; *p* < 0.001]. With respect to gender, males performed significantly better than females in MRT [*t*(181) = −3.80, *p* < 0.001] and OPT [*t*(181) = 2.40, *p* < 0.05] and also showed lower reaction times [RTM: *t*(181) = −6.77, *p* < 0.001; RTV: *t*(181) = −2.64, *p* < 0.005]. No significant gender differences were found in overall cognitive functioning and driving measures. Except for Education, all correlations between predictors, and between the predictors and one or more of the four FTD measures, were significant. No correlation coefficients between predictors was higher than.75, suggesting that there were no issues of multicollinearity among them. As a result, all variables were included in multiple regression models.

### Multiple Regression Analyses

A series of multiple regression analyses was run considering the demographic variables (i.e., age group, gender, and education) and the cognitive variables (i.e., MoCA, MRT, and OPT scores) as predictors of the driving measures. Four separate couples of multiple regression models were performed, one for each of the driving subtests as outcome (e.g., DT, RTM, RTV, and ATAVT). To start, models with independent predictors (main effects) were tested. Subsequently, interactions (first order effects) between the two class of predictors were added. For each of the driving outcomes the fits of the two models were compared with a series of ANOVAs and the most parsimonious one was retained and interpreted.

### Determination Test (DT)

The first multiple regression analysis was performed on DT subtest. Significant and non-significant effects are reported in Table 2. *R*^2^ for the main effect model was equal to 0.49 (adj. *R*^2^ = 0.47), and for first-order interaction model was equal to 0.52 (adj. *R*^2^ = 0.47). No significant difference was found between the two models [*F*(1, 9) = 0.99, *p* = 0.44]. Moreover, models showed almost equal large effect-sizes (Cohen’s *f*^2^ = 0.90). The first-order effects model showed no significant effects of interaction between cognitive and demographic predictors. Consequently, the main effects model appeared to be more parsimonious than the first-order interaction one, and it was retained and discussed. With respect to the main effect model, significant results were found for the effects of age group (β = 0.782; *p* < 0.001), gender (β = −0.242; *p* < 0.05), overall cognitive functioning (β = 0.884; *p* < 0.05), mental rotation (β = 0.205; *p* < 0.05), and perspective-taking abilities (β = −0.120; *p* < 0.005) (see Table 2).

**TABLE 2:** Standardized beta coefficients, Standard Errors and significance levels for predictors for DT variable. Adjusted *R*-squared and *F* statistic are also reported.

	Main effects				Main + first order interaction effects			
	β	Std.Err.	*t*	*p*-value	β	Std.Err.	*t*	*p*-value
**Models’ comparison-determination test (DT)**								
AGE	–0.782	0.111	–7.016	<0.001	–0.736	0.114	–6.445	<0.001
GENDER	–0.242	0.100	–2.409	<0.050	–0.189	0.106	–1.771	0.078
EDUCATION	–0.446	0.314	–1.419	0.157	–0.219	0.347	–0.631	0.528
MoCA	0.884	0.354	2.494	<0.050	0.893	0.398	2.243	<0.050
PT	–0.120	0.036	–3.312	<0.005	–0.103	0.037	–2.273	<0.005
MRT	0.205	0.101	2.022	<0.050	0.203	0.108	1.873	0.062
AGE*MOCA	–	–	–	–	0.142	0.393	0.362	0.718
AGE*PT	–	–	–	–	–0.040	0.041	–0.979	0.329
AGE*MRT	–	–	–	–	0.147	0.117	1.256	0.210
EDUCATION*MOCA	–	–	–	–	1.161	1.194	0.973	0.332
EDUCATION*PT	–	–	–	–	–0.055	0.117	–0.474	0.635
EDUCATION*MRT	–	–	–	–	0.307	0.363	0.845	0.399
GENDER*MOCA	–	–	–	–	0.092	0.404	0.229	0.819
GENDER*PT	–	–	–	–	–0.016	0.038	–0.418	0.676
GENDER*MRT	–	–	–	–	0.063	0.108	0.590	0.555
AdjR^2^	0.475				0.475			
ΔAdjR^2^	0.0001							
F^(*sig.)*^	0.990							

### Visual Reaction Times (RTV)

The second multiple regression analysis was performed on RTV subtest (Table 3). Predictors were the same as in the previous analyses. *R*^2^ of the main-effects model and first-order interaction model were *R*^2^ = 0.16 (adj. *R*^2^ = 0.13) vs. *R*^2^ = 0.21 (adj. *R*^2^ = 0.13), respectively. Models did not differ significantly [*F*(1, 9) = 1.06, *p* = 0.39] and showed medium effect-sizes (Cohen’s *f*^2^ = 0.19 and 0.26, respectively). No significant interaction effects were found. The main effects model was retained.

**TABLE 3:** Standardized beta coefficients, Standard Errors and significance levels for predictors for RTV variable. Adjusted *R*-squared and *F* statistic are also reported.

	Main effects				Main + first order interaction effects			
	β	Std.Err.	*t*	*p*-value	β	Std.Err.	*t*	*p*-value
**Models’ comparison-visual reaction time (RTV)**								
AGE	–0.532	0.178	–2.989	<0.010	–0.506	0.182	–2.780	<0.010
GENDER	0.335	0.161	2.084	<0.050	0.436	0.170	2.560	<0.050
EDUCATION	–0.140	0.502	–0.279	0.780	–0.104	0.554	–0.188	0.850
MOCA	0.777	0.567	1.373	0.171	0.924	0.635	1.456	0.147
PT	0.051	0.058	0.880	0.380	0.067	0.060	1.111	0.268
MRT	0.325	0.162	2.002	<0.050	0.273	0.173	1.583	0.115
AGE*MOCA	–	–	–	–	0.985	0.627	1.571	0.118
AGE*PT	–	–	–	–	0.029	0.065	0.442	0.658
AGE*MRT	–	–	–	–	0.220	0.187	1.176	0.241
EDUCATION*MOCA	–	–	–	–	1.524	1.904	0.801	0.424
EDUCATION*PT	–	–	–	–	–0.026	0.186	–0.142	0.887
EDUCATION*MRT	–	–	–	–	–0.173	0.579	–0.300	0.764
GENDER*MOCA	–	–	–	–	–0.546	0.644	–0.849	0.397
GENDER*PT	–	–	–	–	–0.062	0.061	–1.021	0.308
GENDER*MRT	–	–	–	–	0.128	0.171	0.750	0.454
AdjR^2^	0.133				0.135			
ΔAdjR^2^	0.002							
F^(*sig.)*^	1.070							

Significant results were found for the effect of age group (β = −0.532; *p* < 0.005), gender (β = 0.336; *p* < 0.05), and mental rotation ability (β = 0.325; *p* < 0.05) in the main-effects model (see Table 3).

### Motor Reaction Times (RTM)

The third multiple regression analysis was performed on RTM subtest (Table 3). Predictors was the same as in the previous analysis. *R*^2^ of the main-effects model was *R*^2^ = 0.35 (adj. *R*^2^ = 0.32). *R*^2^ = 0.40 (adj. *R*^2^ = 0.35) emerged for the first order interaction model. Models did not differ significantly [*F*(1, 9) = 1.73, *p* = 0.08] and both showed large effect-sizes (Cohen’s *f*^2^ = 0.53 and 0.67, respectively). In the first-order interaction model, no significant interaction effects were found between the two classes of predictors. As in the previous analysis, the main-effects model has been discussed. Considering the main effects model, significant results were found for the effect of age (β = −0.603; *p* < 0.001) and gender (β = 0.875; *p* < 0.001) (see Table 4). No cognitive predictors were shown to significantly affect the motor reaction times.

**TABLE 4:** Standardized beta coefficients, Standard Errors and significance levels for predictors for RTM variable. Adjusted *R*-squared and *F* statistic are also reported.

	Main effects				Main + first order interaction effects			
	β	Std.Err.	*t*	*p*-value	β	Std.Err.	*T*	*p*-value
**Models’ comparison-motor reaction time (RTM)**								
AGE	–0.603	0.152	–3.949	<0.001	–5.994	0.153	–3.904	<0.001
GENDER	0.875	0.138	6.336	<0.001	0.897	0.144	6.239	<0.001
EDUCATION	–0.244	0.430	–0.568	0.570	–0.198	0.467	–0.424	0.672
MOCA	0.436	0.485	0.898	0.370	0.446	0.535	0.834	0.405
PT	–0.052	0.049	–1.056	0.292	–0.044	0.051	–0.872	0.384
MRT	0.133	0.138	0.959	0.338	0.220	0.145	1.515	0.131
AGE*MOCA	–	–	–	–	0.642	0.529	1.214	0.226
AGE*PT	–	–	–	–	–0.060	0.055	–1.087	0.278
AGE*MRT	–	–	–	–	0.137	0.158	0.871	0.385
EDUCATION*MOCA	–	–	–	–	2.423	1.606	1.502	0.134
EDUCATION*PT	–	–	–	–	0.061	0.157	0.389	0.698
EDUCATION*MRT	–	–	–	–	0.365	0.488	0.749	0.455
GENDER*MOCA	–	–	–	–	0.106	0.543	0.197	0.844
GENDER*PT	–	–	–	–	–0.033	0.051	–0.654	0.514
GENDER*MRT	–	–	–	–	–0.285	0.144	–1.974	0.050
AdjR^2^	0.325				0.349			
ΔAdjR^2^	0.024							
F^(*sig.)*^	1.730							

### Traffic Tachistoscopic Visual Acquisition (ATAVT)

Table 5 shows the multiple regression analysis performed on the ATAVT subtest. Predictors were the same as in the previous analyses. *R*^2^ were equal to 0.27 (adj. *R*^2^ = 0.25) for the main effects and *R*^2^ = 0.35 (adj. *R*^2^ = 0.29) for the interaction effect. Each model showed large effect-sizes (Cohen’s *f*^2^ = 0.38 and = 0.53, respectively) and a significant difference [*F*(1, 9) = 2.08, *p* < 0.05]. In the main-effects model, significant results were found for the effect of age group (β = −0.645; *p* < 0.001), and cognitive functioning (β = 1.71; *p* < 0.005) on Traffic Tachistoscopic Visual Acquisition. These two main effects remained significant even after the insertion in the analysis of first-order interactions. Moreover, a significant interaction effect gender group^∗^perspective taking ability (β = −0.166; *p* < 0.005), was found (see Table 5). The last result suggested that there were no differences in performance between male and female participants at lower levels of ability in OPT; on the contrary this difference increased at higher levels of ability in OPT, in favor of the male. The first order interaction model was discussed.

**TABLE 5:** Standardized beta coefficients, Standard Errors and significance levels for predictors for ATAVT variable. Adjusted *R*-squared and *F* statistic are also reported.

	Main effects				Main + first order interaction effects			
	β	Std.Err.	*t*	*p*-value	β	Std.Err.	*t*	*p*-value
**Models’ comparison-obtaining an overview (ATAVT)**								
AGE	–0.654	0.178	– 3.620	<0.001	–0.676	0.178	– 3.808	<0.001
GENDER	–0.737	0.161	–0.457	0.648	0.044	0.166	0.266	0.790
EDUCATION	–0.277	0.502	–0.533	0.581	–0.894	0.540	– 1.655	0.099
MOCA	1.712	0.566	3.022	<0.010	1.796	0.619	2.899	<0.010
PT	–0.113	0.058	–1.945	0.053	–0.072	0.059	– 1.218	0.225
MRT	0.158	0.162	0.974	0.331	0.089	0.168	0.527	0.599
AGE*MOCA	–	–	–	–	–0.023	0.612	–0.038	0.969
AGE*PT	–	–	–	–	–0.016	0.064	–0.262	0.793
AGE*MRT	–	–	–	–	0.100	0.183	0.548	0.584
EDUCATION*MOCA	–	–	–	–	–0.633	0.858	–0.341	0.733
EDUCATION*PT	–	–	–	–	0.209	0.182	1.146	0.253
EDUCATION*MRT	–	–	–	–	–0.498	0.565	–0.882	0.379
GENDER*MOCA	–	–	–	–	0.136	0.628	0.217	0.828
GENDER*PT	–	–	–	–	–0.166	0.059	–2.782	<0.010
GENDER*MRT	–	–	–	–	–0.049	0.167	–0.294	0.769
AdjR^2^	0.251				0.290			
ΔAdjR^2^	0.039							
F^(*sig.)*^	2.080*							

*p < 0.05.

## Discussion

The present study aimed to investigate the role of cognitive variables (i.e., overall cognitive functioning and spatial mental transformations) and demographic variables (i.e., age, gender, and education) as predictors of fitness to drive in a sample of younger and older adult active drivers. Results showed that: (i) age negatively affects all driving measures considered in this study; (ii) measures of overall cognitive functioning significantly predicted traffic stress resilience and the ability to manage an overview of the traffic visual scene; (iii) measures of object-based spatial mental transformation significantly predicted the performance in both stress resilience and visual reaction task; (iv) significant effects of self-based spatial skills were found on stress resilience and, in interaction with the gender, on visual acquisition; and (v) the interaction between cognitive and demographic predictors mitigated in a non-significant way the effects of these predictors only on measures of stress resilience and speed reaction but not on measures of motor speed and visual acquisition.

The background research approaching individual differences in driving performance demonstrated that aging affects driving skills due to decline in sensory, cognitive, and motor functioning (e.g., Matthews et al., 1999; Mathias and Lucas, 2009; Fraade-Blanar et al., 2018; Kunishige et al., 2019; Ledger et al., 2019a). The current study supports previous findings in which gender differences were related to differences in stress vulnerability (Matthews et al., 1999) and in reaction times (Matthews et al., 1999; Der and Deary, 2006; Dykiert et al., 2012). Regarding education, previous research showed that level of education was not related to vehicle-crash involvement when compared to other variables, such as annual mileage (Lourens et al., 1999). In this study, the two age groups showed a significant difference in terms of levels of education, although in none of regression’s models did the level of education give a significant contribution in predicting the driving prerequisites, and it did not modify the contribution of age in predicting driving abilities.

In addition to demographic predictors, both overall cognitive functioning and spatial mental transformation skills proved to be influential on fitness to drive.

No significant interaction effects were found on measures of traffic stress resilience and reaction tasks; thus, the main effects models were discussed for these outcomes. A significant effect of interaction between self-based spatial mental transformation and gender was found on measures of visual acquisition and therefore the first-order’s interaction model was retained and discussed for this outcome.

### Traffic Stress Resilience

Age, gender, overall cognitive functioning, and both measures of spatial mental transformation significantly predicted traffic stress resilience. Except for education, all predictors employed in this model significantly affected the performance in the task of stress resilience. In the Determination Test, psycho-physical stress and irritating feelings were elicited by using a high frequency of stimuli (DT; Schuhfried, 1998; Schuhfried, 2014). This test provided a valid measure of stress-resilience, typical of the most motivated participants (Hoyos, 1960; Kisser et al., 1986). The significant effect of age suggested that, with the increase of age, drivers became more vulnerable to traffic-stress. Furthermore, females showed better performance than male participants, maintaining an accurate pattern of response under stress conditions. This result is in line with previous findings that highlighted gender differences in favor of males in simple reaction tasks but not in complex reaction task (Dykiert et al., 2012). Matthews et al. (1999) underlined the effects of age and gender on stress susceptibility during driving performance. The authors showed that older people were more vulnerable to stress than younger people. Stress vulnerability resulted in an impairment of vehicle control. Previous research has examined the influence of the driver’s demographic characteristics on safe driving measures, using factors such as collision rates (see Langford et al., 2006). Results from the present study extended this evidence, suggesting age and gender differences in driving tasks that comprise the ensemble of prerequisites to FTD. The execution of DT captures cognitive processes underlying some driving subtasks in the real world, as in high traffic roads where multisensorial stimuli requires continuous monitoring, giving accurate and instantaneous responses. It seems that gender and age-related cognitive changes significantly affected the capability to cope with stress while driving in adults. This also suggests the usefulness of a DT test for the evaluation of driving fitness even in middle-aged drivers.

The significant effects of overall cognitive functioning, Mental Rotation, and Perspective Taking abilities on traffic stress resilience suggests some considerations. Firstly, MoCA total score was shown to be a predictive measure of resilience to traffic stress which, in turn, proved to be highly effective in predicting overall on-road driving performance (Vetter and Debelak, 2012). This result confirmed the effectiveness in performing global cognitive measures in the FTD assessment (Kwok et al., 2015). Considering the age trajectories of cognitive functioning and crash involvement they showed inverse trends: crash rates increased with the decrease of cognitive functioning.

What about this relationship during middle-age? A *subtle cognitive decline* is one of the markers of preclinical Alzheimer’s Disease (Sperling et al., 2011; National Institute on Aging and Alzheimer Association, NIA-AA), an asymptomatic stage of the disease in which people are classified as cognitively normal (Edmonds et al., 2015). Cognitive screening tools, sensitive in discriminating healthy subjects from those with mild cognitive impairment and dementia, need to be validated for FTD purposes. This become more important since changes in cognitive functioning and driving performance occur in middle-age people (Salthouse, 2009; Svetina, 2016). Several scholars showed that measures of mental status predicted on-road and simulated driving measures (for a review see Anstey et al., 2005). A recent study found comparable findings by using the Mini-Mental State Examination (MMSE) and by comparing simulated driving measures and overall cognitive functioning in younger and older drivers (Ledger et al., 2019a). The few studies that have investigated the relationship between MoCA score and driving measures showed inconsistent results. Kwok et al. (2015) investigated the predictive value of MoCA on real-world driving. The authors found a moderate predictive validity on driving performance (i.e., cut-off ≤ 25; sensitivity = 84.5%; specificity = 50%), concluding that MoCA cannot be used as the sole instrument for the identification of unfit drivers. Koppel et al. (2013) investigated the relationship between cognitive functioning and on-road driving behavior in older drivers by using the MoCA score and found no significant results.

Moreover, the ability to react under traffic stress condition, which involves a large amount of distinct cognitive resources, was predicted by specific measures of spatial mental transformation skills. The Determination Test assesses the ability to select appropriate response patterns in a task and to employ visual and acoustic discrimination, and measures psychomotor speed and sustained and selective attention.

Both measures of self-based and object-based spatial mental transformation significantly predicted traffic stress resilience. Although a number of scholars have underlined the key role of visuospatial abilities in fitness-to-drive (Ball and Owsley, 1993; Sommer et al., 2008; Ranchet et al., 2012; Vetter et al., 2018), measures of spatial mental transformation abilities were scarcely accounted for in these studies. Several tools were used to assess the relationship between visuo-spatial functioning and driving performance. In a meta-analysis, Mathias and Lucas (2009) recognized as the most employed tools: The Useful Field of View (UFOV, Ball et al., 1988), the Rey-Osterrieth Complex Figure Test (RCFT Copy and Recall, Rey, 1941), the Trail-Making Test (TMT part A and B; Partington and Leiter, 1949), and the Paper-Folding Test (PFT; Ekstrom et al., 1976). The Paper-Folding Test (PFT) is unique among these visuospatial tests in assessing object-based spatial mental transformation ability, showing the strongest association with measures of driving performance (Anstey et al., 2005). Measures of PFT also showed negative correlations with collision rates and a positive relationship with safe driving both in older and younger drivers (De Raedt and Ponjaert-Kristoffersen, 2000; Andrews and Westerman, 2012). Unlike the measures of object-based spatial mental transformation considered here (i.e., the mental rotation), the PFT cannot be solved by rigid rotations of the visual stimulus (Shepard and Feng, 1972; Ekstrom et al., 1976; Kozhevnikov and Hegarty, 2001). The present results were consistent with the above-mentioned findings on the role of object-based spatial manipulation ability in driving fitness, also suggesting the extension to the role of self-based spatial manipulation skills in predicting some aspects of FTD.

Previous findings demonstrated that both mental rotation and perspective-taking abilities showed a positive relationship with environmental learning. On one hand, mental rotation predicted orientation and wayfinding abilities in the real world (Malinowski, 2001; Ishikawa, 2019), on the other hand the spatial perspective-taking was considered essential for the effectiveness of environmental encoding (Kozhevnikov et al., 2006; Ruginski et al., 2019). Recently, Ruginski et al. (2019) showed that the use of a GPS negatively affects spatial learning trough the mediation of spatial mental transformation skills. Therefore, the present results suggest that both Mental Rotation and Perspective Taking abilities could prove to affect safe driving by (i) reducing hazardous maneuvers; (ii) making the drivers more confident in vehicle-control; and (iii) supporting the spatial orientation ability (Ruginski et al., 2019), especially in stressful driving situations. Finally, given that changes in spatial cognition are considered early neuropsychological markers of Alzheimer’s Disease (Boccia et al., 2019), the assessment of Mental Rotation and Perspective Taking skills could be useful in the evaluation of driving fitness and, in turn, in the car’s accident prevention.

Overall, the present findings were consistent with previous research (Matthews et al., 1999; Der and Deary, 2006; Dykiert et al., 2012) that age negatively affects a driver’s capability to cope with stressful driving circumstances. Unlike the results found by Matthews et al. (1999), female participants in this study showed greater resilience to traffic stress than males. These differences could derive from the different driving tasks and simulators employed. Moreover, these results also suggested specific contributions of measures of overall cognitive functioning, object, and self-based spatial mental manipulation skills in predicting resilience to traffic stress.

### Reaction Times

Significant results were found considering the main effects of Age and Gender on Motor Reaction Times (i.e., motor speed), and the effects of Age and Mental Rotation on Visual Reaction Times (i.e., speed reaction). Overall, these results confirmed those found in previous research, highlighting gender differences in reaction times that become longer and more erratic with the increase of age (Kaber et al., 2012; Leversen et al., 2013; Dickerson et al., 2014; Svetina, 2016). The results for the main effects models were discussed separately for each outcome.

#### Visual Reaction Time

Significant effects were found in the main effects model on speed reaction (i.e., Visual Reaction Times; RTV) by Age and Mental Rotation abilities. The relationship between age and speed reaction times has been previously investigated, with studies mainly highlighting a slowing of response with the increase of age (Birren et al., 1962; Eckert et al., 2010; Svetina, 2016). The age-related changes in speed processing have been attributed to structural and functional decline in the prefrontal cortex (i.e., attentional related neural system; Schiavone et al., 2009), sensory cortex (Salthouse, 2000), and cerebellar gray matter (Rodrigue et al., 2005). Eckert et al. (2010) investigated the neural network of structural-based changes underlying the slower speed processing observed with age in a sample of 42 people between 19 and 79 years of age. Authors found a pattern of cerebellar gray and white matter associated uniquely to age-related decline in perceptual and motor processing performance. In the study of Andrews and Westerman (2012), older drivers showed longer reaction times in complex reaction tasks but not in simple reaction tasks. Older drivers also exhibited significantly poorer performances in tests of psychomotor speed and logical reasoning than younger drivers (Andrews and Westerman, 2012). Considering the effect of age, a similar result was found in the study of Svetina (2016) in which the relationship between age and reaction speed was found to be linear from the age of 20–80. Age-related changes in reaction times were progressive throughout the driver’s lifespan and changes in driving performance could occur before the age of 65.

Mental Rotation abilities influenced the Visual speed measures employed here. This result probably emerged because the nature of the visual reaction measure which involves (at least some of) the object-to-object representation processes underpins the execution of Mental Rotation test (i.e., spatial visualization; Kozhevnikov and Hegarty, 2001). Andrews and Westerman (2012) found that higher spatial visualization ability, measured by the Paper Folding Test (Ekstrom et al., 1976), was significantly associated with shorter headway adoption and less variable lane position by using a simulated driving task in a sample of adults. This suggested that the cognitive resources underling the execution of Mental Rotation and the Paper Folding-Test might share common spatial visualization processes (i.e., 3-D manipulation) and speed processing together with some driving visual subtasks (i.e., track the pedestrian’s movement; Anstey et al., 2005).

#### Motor Reaction Time

A significant effect of age and gender on motor speed was found (i.e., Motor Reaction Times; RTM). Older people and women showed longer RTM than young adults and men. Gender differences in motor coordination abilities have been well documented since childhood (Sanders and Kadam, 2001). Moreover, in the study by Dykiert et al. (2012), male participants showed shorter times in simple reaction tasks than female but not in complex reaction tasks. Tasks that require aiming at static or moving objects seemed to advantage males (Auyeung et al., 2012), while females showed better performances in tasks of fine motor dexterity (Nicholson and Kimura, 1996). In their study, Ashok et al. (2016) found shorter motor times in brake responses in males than females, partially confirming results of a previous study (Warshawsky-Livne and Shinar, 2002). These gender differences in motor response times were attributed to: (i) the presence of more muscle fibers that allow males to quickly respond (Narhare et al., 2012); (ii) differences in neural processing speed (Adam et al., 1999); and (iii) differences in nerve conduction speed due to sex-steroid (i.e., testosterone exposure marked by 2D:4D digit ratio), that allow different neurotransmitter availabilities and spike conduction (Reed et al., 2004; Namita and Shenvi, 2010). Finally, according to Hancock et al. (2002) females tend to avoid, by self-regulating, highly demanding stress situations. This makes them at-risk in sudden hazardous on-road situations.

### Obtain an Overview of Traffic

In the main effects and in the first order interaction models, both age and cognitive functioning significantly predicted the ability to obtain an overview of traffic visual information. The first order interaction model also revealed a significant effect of interaction between Perspective Taking and Gender.

Results showed that an increase in age corresponds to a decrease in the ability to obtain quickly an overview, that is, the ability to quickly detect visual information from a traffic scenario (e.g., information about pedestrians, traffic signs, traffic lights, others vehicle, etc.). Moreover, in both age groups, an increase of the overall cognitive functioning saw performance increase. This result was consistent with previous findings that showed perceptual and cognitive decline in older drivers (for a review: Mathias and Lucas, 2009) and suggest that the MoCA total score easily detects cognitive domains sustaining the fitness to drive.

The effect of interaction between OPT performance and gender suggested that men were better-suited than women at self-based spatial skills in solving a visual perception task. In the study by Zacks et al. (2002), no gender differences were found in Perspective Taking abilities. According to Xistouri and Pitta-Pantazi (2006), males showed systematically higher scores than women. This result provided additional information for the assessment of driving fitness, suggesting specific gender differences in displaying abilities relevant for safe driving.

The ATAVT test is described as a measure of an individual’s visual attention, visual perception, and perceptive speed that are considered valid predictors of crash involvements (e.g., Sims et al., 1998) and accident rates (e.g., Owsley et al., 1994), especially in older drivers. For example, Vetter et al. (2018) demonstrated the high predictive value of the ATAVT test on measures of on-road-driving performance in a sample of experienced professional drivers. A recent review highlighted the detrimental effect of aging on visual perception (Woutersen et al., 2017), also showing neurophysiological evidence that older drivers are more prone to deeply process irrelevant stimuli (Hahn et al., 2013). In other studies, older drivers showed impairments in both accuracy and speed of understanding traffic signs (Stutts et al., 1998; Ben-Bassat and Shinar, 2015; Schulz et al., 2019). The reduced comprehension of traffic signs was found to be mainly associated with age-related changes in selective attention, speed perception, and semantic memory (Lesch et al., 2013; Boot et al., 2014; Toepper et al., 2014).

Overall, these results highlighted the significant effects of Age and Cognitive functioning in predicting the visual acquisition of traffic-related information, also suggesting the contribution of gender in influencing the role of self-based spatial mental transformation skills in FTD.

## Conclusion

Taken together, the results of the present study provided a coherent framework of information highlighting the central role of age in predicting measures of fitness to drive.

Traffic stress resilience was shown to be negatively affected by age and positively affected by overall cognitive functioning, gender, and spatial skills. Reaction times were positively influenced by gender (motor reaction only) and mental rotation skills (visual reaction only), and negatively affected by age. Perceptual speed as a measure of the ability to obtain an overview was negatively affected by age and positively affected by cognitive functioning and an interaction between gender and perspective-taking skills. The assessment of these driving prerequisites in different driving groups could take into account the results presented here.

To the best of our knowledge this is the first study to assess the relationship between spatial mental transformation skills and measures of fitness to drive, providing interesting insights on the relationship between mental representations of external space and driving performance. The specific role of spatial transformation skills in predicting prerequisites for FTD beyond the individual’s general intelligence was considered.

From a practical point of view, the installation of cameras and monitors into cars is becoming increasingly frequent with the aim to aid drivers both in navigation (e.g., GPS-based devices) and in detecting visual scenarios beyond their own field of vision (Teranishi et al., 2019), facilitating complex maneuvers such as parking. These systems provide objective viewpoints of scenes that are unavailable from a subjective-egocentric point of view during driving. The use of these aids requires visualization and decoding both categorical and coordinate spatial relations from the displayed perspectives, involving both mental rotation and perspective-taking mental processes (e.g., Lopez et al., 2020). The results presented here demonstrated that these two spatial mental transformations were also involved in the execution of complex behaviors connected to driving. It is likely that interacting with navigation aid devices while driving represents a problem, among others, for the simultaneous involvement of spatial mental transformation resources used while driving and the decoding of the information provided by GPS navigation devices.

The demographic and cognitive determinants that in this study showed to predict driving measures could be considered for clinical and legal issues, such as license holding, license revision/renewal, or in identifying unsafe drivers. This information could improve the assessment of fitness to-drive, helping professionals with simple and brief standardized tools that provide a safe and affordable alternative to on-road tests.

Future developments could include several theoretical and methodological improvements. First, it would be helpful to include a larger age range (Svetina, 2016). Second, it would be challenging to study cognition together with personality and demographic variables in order to better understanding the complexity of FTD determinants (Sommer et al., 2008). Moreover, other domains could be introduced, making the predictive model more complete and in order to study potential mediation/moderation effects. For example, it would be useful to investigate the role of cognitive reserve in older adults (Stern, 2002; Caffò et al., 2016) or topographical navigation/mental representation abilities (Bocchi et al., 2019; Lopez et al., 2019) to expand the scope of the investigation of visuospatial components in fitness-to-drive issue. These improvements would further enrich the set of tools for the assessment of driving skills that professionals of mobility centers could employ for comprehensive and highly predictive cognitive screening in drivers throughout their lifetime.

## Data Availability Statement

The datasets presented in this study can be found in online repositories. The names of the repository/repositories and accession number(s) can be found below: Open Science Framework—OSF, https://osf.io/6sjw9/. Preregistration link: https://osf.io/apsdh.

## Ethics Statement

The studies involving human participants were reviewed and approved by the Department of Education, Psychology and Communication, University of Bari, Aldo Moro. The patients/participants provided their written informed consent to participate in this study.

## Author Contributions

All authors contributed to idea conception, data extraction and analysis, and to writing the first draft of the Manuscript, contributed to article revision and approval of the final version.

## Conflict of Interest

The authors declare that the research was conducted in the absence of any commercial or financial relationships that could be construed as a potential conflict of interest.
