# Supplementary figures and images for: Spatial Mental Transformation Skills Discriminate Fitness to Drive in Young and Old Adults
Source: Front Psychol. 2020 Dec 3;11:604762. doi: 10.3389/fpsyg.2020.604762 (PMC7745720; doi:10.3389/fpsyg.2020.604762)

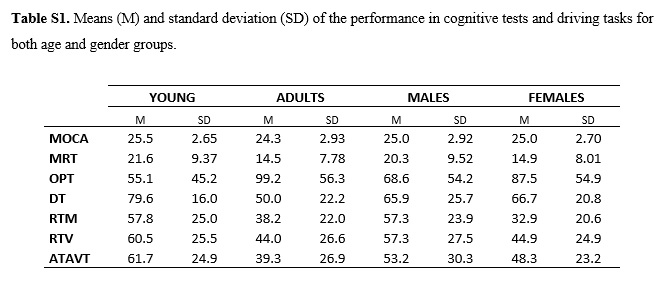

Supplement: Supplementary file 1 [file Image_1.jpg]
